# An Epidemiological Study of Cervical Cancer Trends among Women with Human Immunodeficiency Virus

**DOI:** 10.3390/healthcare12121178

**Published:** 2024-06-11

**Authors:** Arlesia Mathis, Ukamaka D. Smith, Vanessa Crowther, Torhonda Lee, Sandra Suther

**Affiliations:** 1 College of Pharmacy and Pharmaceutical Sciences, Institute of Public Health, Florida Agricultural and Mechanical University, Tallahassee, FL 32307, USA; ukamaka.smith@famu.edu (U.D.S.); sandra.suther@famu.edu (S.S.); 2 Division of Health Care Management, School of Allied Health Sciences, Florida Agricultural and Mechanical University, Tallahassee, FL 32307, USA; vanessa.crowther@famu.edu; 3 Department of Graduate Public Health, Tuskegee University, Tuskegee, AL 36088, USA; tlee2@tuskegee.edu

**Keywords:** cervical cancer, HIV, health disparities

## Abstract

The purpose of this study was to examine cervical cancer among women with Human Immunodeficiency Virus (HIV) and to discuss targeted strategies to reduce the risk of developing cervical cancer. This study used retrospective data from surveillance reports collected between January 2001 and December 2012. Women with HIV/Acquired Immunodeficiency Syndrome (AIDS) were linked through a crosswalk file to Florida’s cancer registry database to determine which women developed cervical cancer during this period. We examined the cervical cancer trends using age-adjusted cervical cancer rates to examine the changes over time; the geographic variations in cervical cancer and HIV across service areas using a Geographic Information System (GIS); and finally, the cervical cancer rates among women with HIV compared with the cervical cancer rates in the general population. The results show that, over time, the cervical cancer rates in women with HIV/AIDS decreased; however, we detected increases in the cervical cancer rates among women in the general population. The findings of this study show that more work is required to address cervical cancer. This growing burden of cervical cancer implies that targeted interventions are imperative to improving the health status of women with cervical cancer. If properly addressed, the potential to reduce and prevent cervical cancer is achievable.

## 1. Introduction

Cancer is the second leading cause of death for women in the United States [1]. The American Cancer Society estimates that approximately 13,820 women in the United States will be diagnosed with cervical cancer in 2024 [2]. Cervical cancer is a disease in which healthy cells on the surface of the cervix change and form a mass of cells that can spread to other tissues [3]. Of those who develop cervical cancer, 4360 women will die from the disease [2]. Most cervical cancer cases result from genital infection with human papillomavirus (HPV) [4,5].

Previous research conducted on the occurrence of cancer among individuals with Human Immunodeficiency Virus (HIV) has mostly been restricted to cancers that are considered Acquired Immunodeficiency Syndrome (AIDS)-defining [6]. Invasive cervical cancer, non-Hodgkin’s lymphoma, and Kaposi sarcoma are considered AIDS-defining malignancies [5]. Prior to modern highly active antiretroviral therapy (HAART), individuals who were HIV-positive were nearly twice as likely to develop cancer at much higher rates than the general population [7]. After the introduction of HAART in 1996, the life expectancy increased for individuals living with AIDS [8,9]; however, these individuals continued to bear a disproportionate burden of illness and disease [10]. Women experience sex-specific cancers (i.e., invasive cervical cancer), which lead to poor clinical outcomes.

Most studies of cervical cancer in women with HIV examine cervical cancer in populations outside the United States [9]. We conducted a literature search using the search terms cervical cancer and women with HIV in the United States in PubMed. This literature search produced only five studies conducted between 2001 and 2024, examining cervical cancer among women with HIV in the United States, and only one examined racial and ethnic disparities. These studies show that many women who are HIV-positive receive suboptimal care. An earlier study conducted by Rahangdale et al. showed that only 62% of women with HIV with an abnormal pap smear had a follow-up pap smear within 12 months [11]. A later study conducted by Barnes et al. focusing on minority and underserved women with HIV found that 46.1% of the women were not screened in the subsequent 15 months [12]. Because women who are HIV-positive have a four-fold increase in cervical cancer risk compared with that of women without HIV, the NIH Office of AIDS Research recommends cervical cancer screening every 12 months for 36 months. If the results are normal, then cervical cancer screening is recommended every three years for women with HIV [13]. These guidelines also include testing for high-risk HPV.

Although there is a significant amount of research examining HPV in the development of cervical cancer, the relationship between Herpes Simplex Virus (HSV) and cervical cancer has not been as widely explored. However, several recent studies have found a link between cervical cancer and Herpes Simplex Virus 2 (HSV-2). Examining the role of HSV-2 in the development of cervical cancer could lead to another route by which to prevent and treat cervical cancer. According to a study published in 2014 by Li and Wen, HSV-2 is associated with cervical cancer. They found that co-infection with HSV-2 and HPV had a higher relative risk (RR) (3.44) than either HSV-2 (2.79) or HPV (2.98) alone [14]. In another study of Mexican women by Bahena-Roman et al., the patients with HSV-2 had a 1.7 times higher risk of having high-risk HPV than the patients who were HSV-2-negative. Moreover, the patients with high-risk HPV had a nine times higher rate of active HSV-2 infection [15]. Furthermore, a study of HSV-2 infection by Martin-Luther et al. suggested that HSV-2 may induce the inflammation of the cervix, which acts as a co-factor in the formation of cervical cancer [16]. The previous studies examining patients with HIV have found that women with HIV are four times more likely to develop cervical cancer when compared with those women who do not have HIV.

This study adds to the literature by using population-level data gathered from HIV/AIDS surveillance reports and the Florida Cancer Registry to characterize the incidence of cancer among women who were reported to be HIV-positive in Florida between 2001 and 2012. Identifying the areas of increasing HIV and cervical cancer rates can be used to make recommendations on appropriate programmatic inventions for women who are HIV-positive to receive timely and adequate care.

## 2. Materials and Methods

### 2.1. Data Description

This study used retrospective data to examine the changes in cervical cancer among women with HIV. We obtained surveillance reports from the Florida Department of Health, identifying women diagnosed with HIV/AIDS between January 2001 and December 2012 within the state. This study included 15,159 women over the age of thirteen who received a medical diagnosis of HIV or AIDS during the specified time period. The subjects were linked by the Florida Department of Health through a crosswalk file to the Florida Cancer Registry database using social security numbers and dates of birth to determine which of these women also developed cervical cancer. To maintain the confidentiality of the participants, patient identifiers were then removed before the data were provided to the researchers via a data use agreement. The variables gathered from the databases included age, race, age at diagnosis, mode of exposure, and geographic region. The eleven geographic regions used for this study were existing regions designated by the Florida Department of Health for purposes of data collection and service provision. A region can range in size from 1 to 14 contiguous counties; however, most contain 5 or fewer counties. In addition to geographic and demographic information, we also gathered information on the facility where diagnosis was conducted, as well as the education level and insurance type of each of the participants. Education and insurance information was not used in our analysis because of the large percentages of missing data.

In addition to evaluating the development of cervical cancer in women with HIV, we also assessed which women were diagnosed with HSV-2 (Herpes Simplex Virus 2) infection. HSV-2 is transmitted through sexual contact and can increase the risk of HIV infection by two to four times by providing direct contact with blood. We collected data on the number of women diagnosed with HSV-2 during the specified time period. HSV-2 infections were chosen because of the relationship between HSV-2 and HIV. HSV infection can cause sores or breaks in the skin or lining of the mouth, vagina, and rectum, which provides a way for HIV to enter the body. Even without visible sores, HSV-2 increases the number of immune cells in the lining of the genitals, allowing for easier entry into the body. Having both HIV and genital HSV-2 increases the chance of spreading HIV to an HIV-negative partner during oral, vaginal, or anal sex.

In our final analyses, the women with HIV who developed cervical cancer were then compared with the women without HIV diagnoses who developed cervical cancer in the same geographic region during the same time period from a third data resource named the Florida Health Community Health Assessment and Resource Tool Set (CHARTS). Florida Health CHARTS (https://www.flhealthcharts.gov/charts/) (accessed on 18 August 2022) is a website providing online access to health indicator data at the community and statewide levels for the state of Florida.

### 2.2. Data Analysis

We conducted the following analyses to examine cervical cancer in women with HIV. First, we assessed the number of women with HIV or AIDS who developed cervical cancer. The subjects were matched and linked through a crosswalk file to the Florida Cancer Registry database using social security numbers and dates of birth to determine which of these women also developed cervical cancer. We calculated percent differences by race and ethnicity in women who had HSV-2 or developed cervical cancer (see Table 1), and then examined the trends in cervical cancer rates. Cervical cancer rates were calculated using the number of women who were reported to be HIV-positive and who were diagnosed with cervical cancer between 2001 and 2011.

We were also interested in examining whether women with HIV may also have contracted additional sexually transmitted infections. We selected HSV-2 because of the relationship between HSV-2 and HIV. Then, the percentage of women who developed HSV-2 was calculated based on race and ethnicity (see Table 1). Further, we examined the percentage of women in our study who had HIV only, HIV and HSV-2, HIV and cervical cancer, and cervical cancer and HSV-2 (see Table 2).

For our second analysis, we examined the geographical locations of the women with HIV and cervical cancer to determine whether there was a relationship between higher rates of HIV and cervical cancer. High rates of both in a geographic area may be related to poor access to appropriate primary care.

For our third analysis, we compared cervical cancer in the women with HIV against cervical cancer in the women without HIV. We conducted this analysis to determine whether the cervical cancer trends in the women with HIV were the same or different than those in the women without HIV. We selected generalized estimating equations (GEEs) to analyze these data because the GEE tool was developed to analyze longitudinal, nested, or repeated measures designs. The GEE approach was developed by Liang and Zeger in 1986 to produce more efficient and unbiased regression estimates for use in analyzing longitudinal designs with non-normal distributions and is used widely today in epidemiology, gerontology, and biology [17]. Using GEEs, we compared the changes in cervical cancer rates among the women with HIV to those without HIV.

## 3. Results

### 3.1. Characteristics of Populations of Interest

Table 1 illustrates the number of women with HIV/AIDS, cervical cancer, and HSV-2 diagnosed between 1 January 2001 and 31 December 2012. A higher percentage of HIV diagnoses was observed among Black women (70.9%) compared with White (14.1%) and Hispanic women (12.4%). Similar results were also noted in the number of women with cervical cancer. A higher percentage of cervical cancer was detected among Black women, at 69.9%, followed by White and Hispanic women, at 20.4% and 9.7%, respectively. Overall, this showed that more Black women than White or Hispanic women were diagnosed with HIV and cervical cancer.

### 3.2. Changes in Cervical Cancer Rates Based on Age

Table 2 shows the changes in the age-adjusted rates of cervical cancer among the women with HIV based on age group from 2001 to 2011. A chi-square test for trends was conducted to evaluate the changes within each age group over time. There was a significant reduction in cancer rates in the women between the ages of 20 and 29 years old (*p* < 0.01). The changes occurring in other age groups were not significant. For individuals within the age range of 13–19, there were no reported cases of cervical cancer. The highest rate of cervical cancer occurred for women between the ages 30 and 39 and 40 and 49.

### 3.3. Geographic Variations in HIV and Cervical Cancer

**Figure 1:**
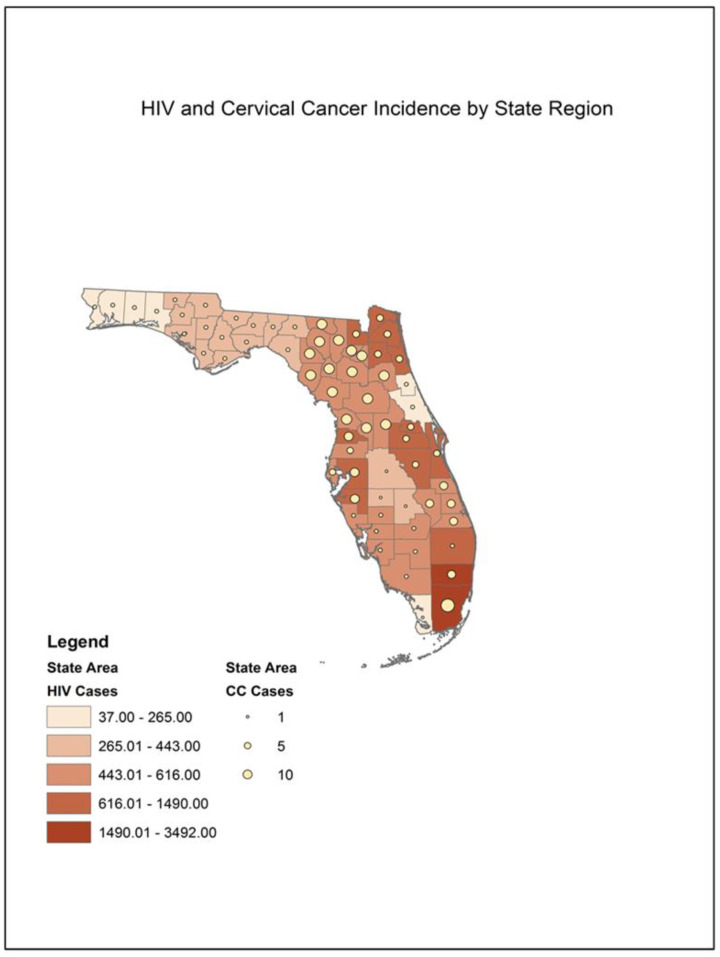
Geographic variation in HIV and cervical cancer. Note: this GIS map shows the number of HIV and cervical cancer cases within each service area.

Figure 1 presents a Geographic Information System (GIS) map that shows the relationship between HIV and cervical cancer based on area. The geographic area was based on regions designated by the Florida Department of Health for the purposes of data collection and service provision. The data are shown based on the number of cases that occur within each region. HIV is designated by color shading on the map, with the darker colors showing higher numbers of HIV cases. Cervical cancer is designated by dot size, with larger dots signifying more cases. Our map shows that the relationship between HIV cases and cervical cancer varies based on this region.

### 3.4. Geographic Variations in HIV and Cervical Cancer

Table 3 shows the occurrence of HSV-2 infection within the population. Of the total number of women who were diagnosed with HIV during the time period of interest, 3.1% were also diagnosed with HSV-2 infection, and 0.6% developed cervical cancer. A small percentage of the women (0.03%) had HIV, HSV-2, and cervical cancer.

Because this research revealed decreases in the rates of cervical cancer, we were interested in examining whether these decreases also occurred among women who also had an HSV-2 infection. As stated earlier in this article, the diagnosis and treatment of HSV-2 infection are important because genital HSV-2 increases the chance of spreading HIV to an HIV-negative partner during sexual contact.

Table 4 shows the changes in the rates of HSV-2 among the women with HIV based on age group from 2001 to 2011. A chi-square test for trends was conducted to evaluate the changes within each age group over time. There was a significant reduction in HSV-2 rates in all the age groups, except the individuals aged between 13 and 19 years. As noted in the table, the rates for the individuals between 13 and 19 appear higher than those in the other groups; however, the rates are based on a smaller population of women.

**Figure 2:**
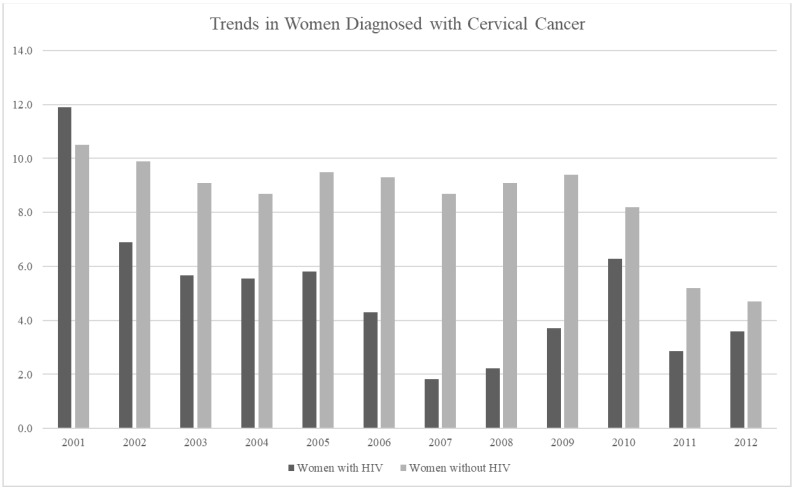
Trends in Florida women diagnosed with cervical cancer. Note: this figure shows a comparison of cervical cancer rates between women with and without HIV.

The researchers conducted a final analysis because the result that showed decreasing rates of cervical cancer within the HIV-positive population was unexpected. To determine if the rates of cervical cancer were really decreasing and confirm that this result was not an artifact of overall decreases in cervical cancer throughout the state, the researchers compared the rates of cervical cancer in women who were reported to be HIV-positive within this study with those without HIV during the same period. The cervical cancer rates for the general population of women were gathered from the Florida Health CHARTS, as shown in Figure 2.

Table 5 shows the results of our final analysis, which examines the trends in cervical cancer between women with and without HIV. We used generalized estimating equations (GEEs) for this analysis. As mentioned earlier, a GEE is a statistical method used widely in the medical and health sciences for modeling longitudinal data [17]. In this study, we examined the changes in cervical cancer rates over time. Our analysis shows a significant difference between the groups.

## 4. Discussion

The findings of this study highlight that women with HIV have decreasing rates of cervical cancer when compared with women without HIV. The previous studies of women who were reported to be HIV-positive with cervical cancer showed mixed results [18,19]. However, when women with only HIV were examined, the changes in cervical cancer rates varied based on age group. For instance, there was a significant decrease in the number of cervical cancer cases in the women who were reported to be HIV-positive between the ages of 20 and 29 years old. This may be attributed to increased access to retroviral therapies. When examining other infections, such as HSV-2 infection among the women with HIV, there were significant changes in all the groups, except the women between 13 and 19 years old. This may be attributed to the smaller number of cases of women with HIV in this age group, which results in a higher infection rate even though the numbers are actually lower. However, among the women between 20 and 29 years old, there was a significant decrease in HSV-2 infection rates observed in this group.

When examining the data geographically, the relationship between HIV cases and cervical cancer varies based on region. Some regions of the state show higher numbers of cervical cancer cases compared with lower numbers of HIV cases. Moreover, the areas with higher numbers of HIV cases vary, showing higher numbers in the northeastern, central, and southeastern parts of the state. The areas with higher numbers of HIV occur in and around larger cities and more densely populated areas; however, higher numbers of cervical cancer appear in the less-populated and rural areas of the state. This shows that there are still areas of disparity, where there may be a lack of health care access among women with HIV. 

This study has several merits that should be mentioned. First, our research findings examined the cervical cancer trends among women who were reported to be HIV-positive over time in one area of the United States. During a literature search on this topic, we found that many of the prior studies examined cervical cancer risk through clinical screening records [20]. We also found that many of these studies examined the cervical cancer risk in women who were reported to be HIV-positive and living outside the United States [21]. The rationale to focus research on women who are HIV-positive living outside of the United States may include the assumption that women in the United States have greater access to health care, and therefore, may have lower rates of cervical cancer than those in other countries. Second, this study included more participants than the clinical screening studies. We collected data through a state surveillance system to examine the cervical cancer rates in over 15,000 women who were HIV-positive over a ten-year period. The HIV and cancer surveillance system captures all the HIV or cervical cancer cases reported within the state at various locations, including clinics, hospitals, physician’s offices, etc. Although this study found lower cervical cancer rates in the women who were reported to be HIV-positive than the previous studies, and the changes over time were significant, it has limitations. Because the data were de-identified to protect the privacy of the women prior to receiving the data for analysis, the researchers were only able to acquire certain reported information for the women in this study. However, by using population-based data, we were able to examine the trends in cervical cancer in a large population of women who were reported to be HIV-positive and living in Florida. Unlike some clinic-based studies, this study was able to include the data of women who were reported to be HIV-positive who may not have received regular care or may have relocated to a different area. Our results were unexpected based on the findings in previous studies. We thought that the cervical cancer rates would be higher in women who are HIV-positive. Our baseline year (shown in Figure 2) did show higher rates of cervical cancer among the women who were HIV-positive; however, the trends in cervical cancer showed that the rates continued to decrease in the women who were HIV-positive, while not showing as much of a decrease in the women without HIV. A possible reason is that the expanded use of highly active antiretroviral therapy reduced the level of infection in women with HIV. Furthermore, women who were HIV-positive in our study may have had more interactions with health professionals, which may increase the likelihood that they received the appropriate preventive services. The HPV vaccination status of the women in our study was not available. However, our GIS map identifies service areas where better access is required.

## 5. Conclusions

Annually, cervical cancer accounts for approximately 364 deaths per year among the women in Florida. This public health issue is of great importance because many types of cervical cancer are preventable. 

Since the introduction of highly active antiretroviral therapy (HAART) in 1996, the life expectancy has increased for people with HIV [22]. Drug combinations lead to decreased viral loads, allowing people with HIV to live longer and healthier lives. Consequently, the population of people with HIV has become more likely to develop other conditions, such as cancers caused by environmental exposure or health-related behaviors [10]. However, it was not until 2010 that a national policy was developed to address this issue [23]. The national policy was updated in July 2015 to measure the outcomes for the next five years and was subsequently updated again in December 2021 [24]. The National HIV/AIDS Strategy provides four goals—(1) to prevent new infections, (2) to improve HIV-related health outcomes of people with HIV, (3) to reduce HIV-related disparities and inequities, and (4) to achieve integrated, coordinated efforts that address the HIV epidemic among all partners and stakeholders.

On 16 July 2013, a Federal Executive Order to establish the HIV Care Continuum Initiative was announced. This initiative identified the need to link people with HIV to health services to maintain care and receive the most therapeutic benefits. Federal, state, local, and community organizations continue to use the HIV Care Continuum Initiative to measure progress toward HIV goals as well as to pinpoint where gaps in services may exist in connecting individuals with HIV to sustained quality care and treatment [25]. There is now a deeper understanding of the long-term care needs of people living with HIV, but further research is required to ensure longevity and quality of life. Our research shows that from 2001 to 2012, there was improvement among women with HIV; however, there continued to be a gap in engagement to care for people living in less-populated areas who may lack access to the required medical services.

### Implications for Policy and Practice

Women who are HIV-positive and are linked to care have better outcomes in disease prevention. The public health implications from our findings suggest that more women require preventive medical services. Although we did not find that the women who are HIV-positive in our study had higher rates of cervical cancer, this study shows that cervical cancer among women in the general population is still problematic. This burden of cervical cancer among women without HIV indicates that targeted prevention and treatment strategies are required. The US Preventative Services Task Force (USPSTF) recommends that all women should begin cervical cancer screening at 21 years of age [26]. However, cervical cancer is mostly found in women who have never been screened or who have not been screened recently. The reasons for not being screened include a lack of health insurance and having no access to preventive services [27]. Our study suggests that higher cervical cancer and HIV infection rates are in areas with fewer service providers. Targeting these areas for health promotion campaigns by local health departments on the importance of cancer screening along with increasing the availability of services through the creation of community health centers or mobile health facilities can make a difference. It will take concerted action by multiple stakeholders to decrease the rates of cervical cancer. If properly addressed, the potential to prevent cervical cancer and other infections is achievable.

## Figures and Tables

**Table 1:** Study population with HIV, cervical cancer, and HSV-2 based on race/ethnicity.

	HIV		Cervical Cancer		HSV-2	
	N	%	N	%	N	%
Race/ethnicity						
White	2142	(14.1)	19	(20.4)	52	(11.1)
Black	10,748	(70.9)	65	(69.9)	349	(74.6)
Hispanic	1878	(12.4)	9	(9.7)	58	(12.4)
Asian	29	(0.2)	0	(0.0)	0	(0.0)
Native Hawaiian/Pacific Islander	2	(0.0)	0	(0.0)	0	(0.0)
Native American	10	(0.1)	0	(0.0)	0	(0.0)
Multi-racial	345	(2.3)	0	(0.0)	9	(1.9)
Unknown	5	(0.0)	27	(29.0)	0	(0.0)
Total	15,159		93		468	

**Table 2:** Changes in cervical cancer rates among women with HIV based on age.

Year	2001	2002	2003	2004	2005	2006	2007	2008	2009	2010	2011	*p*
Ages												
13–19 yrs	0.00	0.00	0.00	0.00	0.00	0.00	0.00	0.00	0.00	0.00	0.00	-
20–29 yrs	4.67	14.02	12.99	8.51	4.95	4.98	0.00	5.46	0.00	0.00	0.00	<0.01
30–39 yrs	21.84	9.71	6.52	8.47	14.81	0.00	4.75	0.00	5.29	10.75	7.49	0.12
40–49 yrs	23.32	7.69	8.83	3.63	2.13	9.11	4.34	2.42	10.20	9.40	6.85	0.62
50+ yrs	9.66	0.00	0.00	7.07	7.17	7.41	0.00	3.17	3.13	11.24	0.00	0.69
Overall	11.90	6.28	5.67	5.54	5.81	4.30	1.82	2.21	3.72	6.28	2.87	0.11

**Table 3:** Cases of HSV-2 and cervical cancer among women with HIV.

	HIV	HIV/HSV-2	HIV/Cervical Cancer	HIV/HSV-2 and Cervical Cancer	Total
Cases	14,603	463	88	5	15,159
Percentage	96.3%	3.1%	0.6%	0.03%	100%

**Table 4:** Changes in HSV-2 rates among women with HIV based on age.

Year	2001	2002	2003	2004	2005	2006	2007	2008	2009	2010	2011	*p*
Ages												
13–19 yrs	17.65	15.38	10.53	0.00	20.00	0.00	5.00	18.75	0.00	0.00	12.50	0.15
20–29 yrs	5.58	7.52	6.61	6.40	2.01	2.30	5.31	1.08	1.25	0.00	0.00	0.03 *
30–39 yrs	4.36	4.17	2.71	2.83	1.48	2.69	2.34	0.54	0.00	0.89	0.93	0.01 *
40–49 yrs	3.13	3.93	2.76	2.59	3.27	1.49	3.05	2.84	1.10	0.79	1.11	0.02 *
50+ yrs	2.89	3.80	2.39	1.59	4.24	0.87	0.44	1.42	0.62	0.65	0.74	0.01 *
Overall	4.04	4.74	3.49	2.97	3.16	1.73	2.51	1.96	0.69	0.63	1.00	0.001 *

Note: Statistically significant *p*-values are marked with an asterisk (*).

**Table 5:** GEE analysis of cervical cancer in women with HIV.

Parameter	β	SE	95% CI		Wald Chi-Square	*p*-Value
			Upper	Lower		
Intercept	2.102	0.13	1.84	2.36	256.28	<0.001

## Data Availability

The data for this study are not publicly available, but may be obtained via a data use agreement from the Florida Department of Health.
